# Stemness regulation in prostate cancer: prostate cancer stem cells and targeted therapy

**DOI:** 10.1080/07853890.2024.2442067

**Published:** 2024-12-23

**Authors:** Hao Liang, Bin Zhou, Peixin Li, Xiaoyi Zhang, Shijie Zhang, Yaozhong Zhang, Shengwen Yao, Sifeng Qu, Jun Chen

**Affiliations:** a Department of Urology, Qilu Hospital of Shandong University (Qingdao), Qingdao, China; b Department of Urology, Qilu Hospital of Shandong University, Jinan, China

**Keywords:** Prostate cancer, cancer stem-like cell, cancer stem cells, tumor heterogeneity, stemness, targeted therapy

## Abstract

**Background:**

Increasing evidence indicates that cancer stem cells (CSCs) and cancer stem-like cells form a special subpopulation of cells that are ubiquitous in tumors. These cells exhibit similar characteristics to those of normal stem cells in tissues; moreover, they are capable of self-renewal and differentiation, as well as high tumorigenicity and drug resistance. In prostate cancer (PCa), it is difficult to kill these cells using androgen signaling inhibitors and chemotherapy drugs. Consequently, the residual prostate cancer stem cells (PCSCs) mediate tumor recurrence and progression.

**Objective:**

This review aims to provide a comprehensive and up-to-date overview of PCSCs, with a particular emphasis on potential therapeutic strategies targeting these cells.

**Methods:**

After searching in PubMed and Embase databases using ‘prostate cancer’ and ‘cancer stem cells’ as keywords, studies related were compiled and examined.

**Results:**

In this review, we detail the origin and characteristics of PCSCs, introduce the regulatory pathways closely related to CSC survival and stemness maintenance, and discuss the link between epithelial–mesenchymal transition, tumor microenvironment and tumor stemness. Furthermore, we introduce the currently available therapeutic strategies targeting CSCs, including signaling pathway inhibitors, anti-apoptotic protein inhibitors, microRNAs, nanomedicine, and immunotherapy. Lastly, we summarize the limitations of current CSC research and mention future research directions.

**Conclusion:**

A deeper understanding of the regulatory network and molecular markers of PCSCs could facilitate the development of novel therapeutic strategies targeting these cells. Previous preclinical studies have demonstrated the potential of this treatment approach. In the future, this may offer alternative treatment options for PCa patients.

## 1. Introduction

Prostate cancer (PCa) is the most common type of malignant tumors of the male genitourinary system. In 2023, it is estimated that 300,000 new cases of PCa will be recorded and >30,000 deaths due to PCa will occur in the United States of America [1]. Age is an important risk factor for PCa [2]. Considering the aging population worldwide, it is expected that the incidence and mortality rates of PCa will increase annually [3]. Patients with early-stage PCa can be treated with radical surgery; however, androgen deprivation therapy (ADT), chemotherapy, or radiation therapy are recommended for patients with relatively advanced PCa, particularly those with multiple metastases [4,5]. ADT has been used for the treatment of PCa for the past 70 years [6]. However, studies have found that most patients with PCa develop drug resistance, and the disease transforms into castration-resistant prostate cancer (CRPC) within 6–20 months after treatment [7].

It is thought that the emergence of drug resistance and transformation of CRPC are related to the tumor heterogeneity of PCa (between patients and within a single tumor), which is ubiquitous in solid tumors [8–10]. Previously, researchers extensively explored the mechanism of CRPC. Numerous studies have shown that alteration of the androgen signaling pathway, as a result of drug treatment, may be the mechanism underlying the occurrence of CRPC. For example, androgen receptor (AR) amplification, AR mutation, and AR aberrant splice variants may drive the progression of CRPC [11,12]. With the in-depth understanding of the mechanism of tumorigenesis and development, increasing evidence indicates that cancer stem cells (CSCs) and cancer stem-like cells play an important role in tumor initiation, progression, recurrence, and metastasis [13,14]. Cancer stem-like cells share similar stem cellular characteristics with CSCs, such as self-renewal, differentiation capacity, and drug resistance. Below, we use CSCs as an umbrella term to describe a subpopulation of cells exhibiting stem cell characteristics within a tumor. Previous studies have suggested that prostate cancer stem cells (PCSCs) play an important role in the progression of PCa [15,16]. In addition, the density and functional status of PCSCs are closely associated with response to treatment and the prognosis of patients with PCa. Considering the prominent role of PCSCs in the regulation of tumor behavior, targeting PCSCs is a promising therapeutic strategy [17].

In this review, we discuss the characteristics, source, function, and regulatory or transformation mechanism of PCSCs, as well as the current research progress and clinical application of drug therapies targeting these cells. Finally, we summarize the limitations of current research and discuss the prospects of PCSC therapy.

## 2. CSCs and PCSCs

### 2.1. CSCs

**Figure 1.**
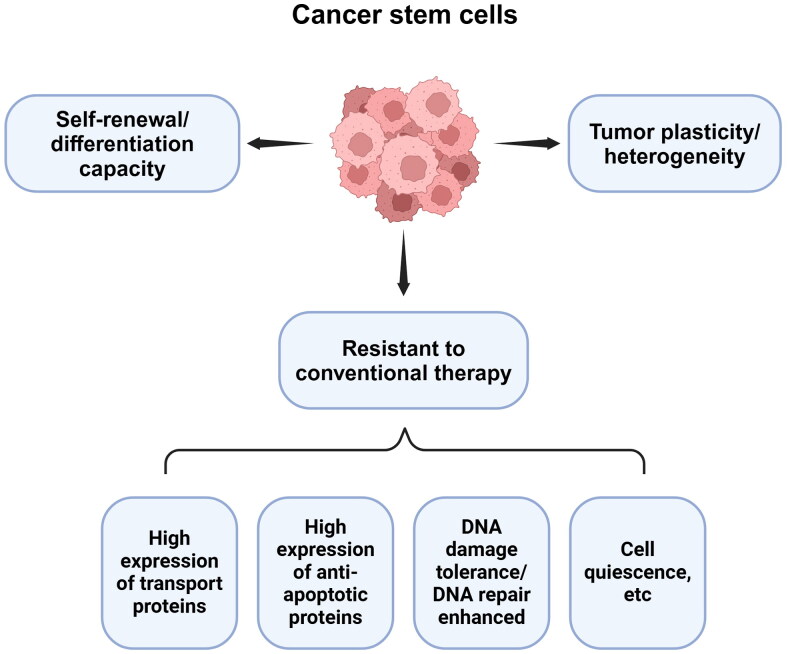
Characteristics of CSCs. CSCs are a subpopulation of cells that are ubiquitous in tumors and have similar characteristics to normal stem cells, namely, self-renewal and differentiation ability. CSCs are a group of dynamically changing cell subsets that can achieve the transformation of stemness and non-stemness under the action of regulatory factors, which reflects the plasticity and heterogeneity of tumors. In addition, compared with common tumor cells, CSCs are more resistant to conventional treatment strategies, which is related to the expression of high levels of transport proteins and anti-apoptotic proteins, enhanced DNA damage tolerance and DNA repair ability, and relative quietness of cells (Created with bioRender.com).

Tumor heterogeneity is a major challenge in the treatment of cancer, and may be related to mechanisms such as gene mutation or epigenetic modification [18]. This causes treatment failure and leads to cancer recurrence and progression. Studies using a CSC model and a CSC plasticity model provided a possible explanation for the generation of tumor heterogeneity [19,20]. Specifically, it was stated that transformation of the cellular state and functional maturation of CSCs or tumor cells with a stem cell phenotype can generate tumor heterogeneity. In addition, the dedifferentiation and trans-differentiation of differentiated tumor cells reflect the plasticity of cells and contribute to the generation of tumor heterogeneity [21]. CSCs have the ability of self-renewal, thereby initiating and maintaining long-term tumor growth, and inter-transforming between non-CSCs and CSC states (as shown in Figure 1). Moreover, CSCs are more resistant to conventional drug therapy (chemotherapy, radiotherapy, molecular targeted therapy, etc.) compared with non-CSC cells. This difference is attributed to the fact that CSCs are in the quiescent phase of the cell cycle [22]. Traditional treatments, such as chemotherapy and radiotherapy, exhibit a satisfactory cytotoxic effect only against rapidly proliferating tumor cells. In addition, CSCs express high levels of transport proteins and anti-apoptotic proteins, and their resistance to DNA damage is responsible for the development of drug resistance [23]. Epithelial–mesenchymal transition (EMT) and mediated angiogenesis are also thought to be associated with drug resistance [24]. Drug resistance of CSCs allows them to survive long-term treatment and promote subsequent tumor recurrence and distant metastasis. This process has been confirmed in numerous studies on solid tumors [25]. The regulatory mechanisms of CSCs are complex and diverse. Several major signaling pathways regulate the survival and development of CSCs, such as the Wnt/β-catenin, hedgehog (Hh), Notch, and phosphatidylinositol 3 kinase/protein kinase B/mechanistic target of rapamycin (PI3K/AKT/mTOR) pathways [26,27]. In addition, various transcription factors, cytokines, microRNAs (miRNAs), and metabolites are involved in the regulation of CSC function [28,29].

### 2.2. PCSCs

Although CSCs were initially discovered in hematological malignancies, they were subsequently reported in a variety of solid tumors. PCSCs were first identified in 2005, providing new directions for research. Further research identified several molecules as markers of PCSCs, such as CD44, CD117, CD133, aldehyde dehydrogenase (ALDH) and ATP-binding cassette transporter protein (ABC) [30]. PCSCs play an important role in the occurrence, progression, and metastasis of PCa [31,32]. However, there is currently a lack of consensus regarding the origin of PCSCs [33,34]. Both luminal progenitor cells and basal stem/progenitor cells in normal prostate tissue may be the source of PCSCs. PCa originating from luminal progenitor cells often manifests as adenocarcinoma, while PCa originating from basal stem/progenitor cells is associated with a more aggressive neuroendocrine phenotype [35–37]. Dedifferentiation and reprogramming of tumor cells are also important for the acquisition of stemness and reflect the plasticity of tumors. Specifically, this plasticity is the ability of tumor cells to switch between CSCs and non-CSC states. Tumor plasticity is important for the generation of tumor diversity or heterogeneity. Of note, significant inter- and intra-tumor heterogeneity has been observed in patients with PCa [38,39]. The intra-tumor heterogeneity of PCa is reflected at various levels (tissue structure, cell morphology, genome, epigenetic modification, metabolic pathways, etc.) [8,40]. Differences in the expression of surface receptors in PCa cells, particularly in the expression levels of AR, are thought to be associated with resistance to anti-androgen therapy. Tumor cells with high expression of AR may die during ADT, whereas those with low expression of AR may survive and proliferate. Unfortunately, the presence of cell populations with low AR expression implies an enrichment of PCSCs [41,42]. The Gleason score is used to evaluate the degree of PCa malignancy in clinical practice. The proportion of PCa cell subsets with no or low expression of AR (AR−/low) in the tumor is related to the degree of malignancy. Researchers found that a greater proportion of the AR−/low subgroup was detected in tumors with G9 versus G7 [43]. Similarly, other studies have demonstrated that the abundance of AR−/low cells in tumors of patients treated with ADT was significantly higher than that recorded before treatment [44,45]. This finding implies that current ADT has an insufficient cytotoxic effect on PCSCs, and new therapeutic strategies targeting PCSCs should be explored. We describe this in more detail below.

## 3. Regulatory mechanisms of PCSCs

The regulatory mechanisms of PCSCs are multilevel and various. Although specific regulatory mechanisms of PCSCs have not been fully elucidated, numerous previous studies have reported important findings. Below, we elaborate on the signal transduction pathways of PCSCs and the effects of EMT regulation of PCSCs on the stemness of PCa and functional status of PCSCs. Finally, we discuss the crosstalk between tumor microenvironment (TME) and PCSCs.

### 3.1. Signaling pathway regulation in PCSCs

#### 3.1.1. Wnt/β-catenin signaling pathway in PCSCs

The Wnt pathway plays an important role in embryonic and organ development by regulating stem cell self-renewal, proliferation, migration, and differentiation [46,47]. In PCa, Wnt signaling is closely associated with the behavior and function of CSCs [48]. The Wnt signaling pathway can be divided into the canonical pathway and non-canonical pathway [49]. In the canonical pathway, Wnt binds to Frizzled receptors and low-density lipoprotein receptor-related protein 5/6 (LRP5/6) co-receptors, resulting in the phosphorylation of LRP5/6 by casein kinase 1 (CK1) and glycogen synthase kinase 3 (GSK3). This is accompanied by the recruitment of axin to the plasma membrane, thereby avoiding β-catenin recruitment and degradation. Subsequently, β-catenin undergoes nuclear translocation and binds to downstream transcription factors and co-stimulatory molecules to regulate the expression of target genes. In the non-canonical pathway, Wnt activates target gene expression and promotes cytoskeleton rearrangement, thus affecting cell adhesion and migration [50]. Additional evidence has shown that activation of Wnt non-canonical signaling promotes resistance to ADT and tumor cell invasiveness in advanced PCa and CRPC [51,52].

Wnt pathway activation enhances the self-renewal and proliferation of CSCs, and promotes the PCSC phenotype by upregulating its markers (i.e. CD44, CD133, ABCG2, ALDH1A) [53–56]. In contrast, downregulation of Wnt/β-catenin signaling using inhibitors or siRNA reduced CSC marker expression and reduced the number of ALDH-positive CSCs [57]. Overall, although the specific regulatory mechanism of Wnt signaling involved in PCSCs remains partly understood, this pathway can be used as a potential therapeutic target against CSCs. Such research may provide new therapeutic options for patients with CRPC.

#### 3.1.2. Notch signaling pathway in PCSCs

The Notch pathway is a highly conserved signaling pathway that regulates cell proliferation, apoptosis, EMT, and drug resistance [58]. Four Notch receptors (Notch1–4) and five Notch ligands (delta like canonical Notch ligand 1 [DLL1], DLL3, DLL4, jagged canonical Notch ligand 1 [JAG1], JAG2) have been identified. Binding of Notch receptors to ligands leads to proteolysis of the Notch receptor transmembrane domain, translocation of the cleavage region of the Notch intracellular domain into the nucleus, and binding to downstream target molecules to form a complex. This process leads to the induction of target gene expression [59]. Notch signaling plays an important role in maintaining CSC populations. Studies have shown that increased levels of JAG1-Notch are associated with the progression, metastasis, and EMT of PCa [60]. Enhanced Notch signaling promotes the expression of multipotency markers, such as CD133, SRY-box transcription factor 2 (SOX2), and octamer-binding transcription factor 4 (OCT4), as well as enhances the ability of cells for spheroid formation [61]. In addition, Notch signaling interacts with nuclear factor-κB (NF-κB), mTOR, Wnt, AR, or transforming growth factor-β (TGF-β) signaling pathways to promote the progression of PCa.

#### 3.1.3. Hh signaling pathway in PCSCs

The Hh signaling pathway regulates cell proliferation, differentiation, and migration, and plays an important role in embryonic organ development [62]. Three Hh ligands, namely sonic hedgehog (SHH), Indian hedgehog (IHH), and desert hedgehog (DHH), bind to the receptor patched (PTCH) to activate the G protein-coupled receptor smoothened (SMO). Activated SMO works with arrestin beta 2 (ARRB2) and kinesin family member 3 A (KIF3A) to promote the transactivation of glioma-associated oncogene homolog (GLI) [63,64], which is involved in the regulation of tumors by targeting gene expression. Aberrant activation of Hh is involved in tumor progression, invasion, and drug resistance. Studies revealed that the Hh pathway is closely linked to the maintenance of CSCs [65,66]. A recent study revealed that regulator of chromosome condensation 2 (RCC2) promotes PCa cell proliferation, metastasis, invasion, and stemness induction of CSCs through Hh/GLI signaling pathway [67]. The expression of Hh pathway component PTCH is significantly higher in metastatic versus primary PCa, and the expression of this molecule is correlated with the pathological grade and stage of disease [68,69]. Furthermore, it has been reported that Hh signaling promotes EMT, thereby potentially contributing to tumor dissemination and transformation to CRPC [70]. Lastly, low AR activity, activation of stemness programs, and Hh pathway were associated with primary androgen signaling inhibitors’ resistance [71].

#### 3.1.4. AR signaling pathway in PCSCs

AR, a member of the steroid receptor superfamily, is a ligand-regulated nuclear transcription factor [72]. It is composed of a C-terminus ligand-binding domain, a DNA-binding domain, and a relatively unstructured N-terminal domain. After binding to ligands, such as testosterone or dihydrotestosterone, dimeric ARs translocate to the nucleus and interact with other regulators to regulate the activation or repression of target genes. AR is mainly expressed in differentiated luminal and stromal cells, exhibiting low expression in basal cells. AR signaling promotes cell differentiation and inhibits excessive proliferation in the normal prostate. In PCa, AR signaling promotes tumor growth and progression. Approximately 98% of PCa cases are adenocarcinomas, with most being composed of AR (+) luminal cells [7]. Therefore, androgen signaling inhibitors have long been the mainstay of treatment for patients with PCa. Nevertheless, as mentioned above, PCa is a highly heterogeneous type of cancer. In addition to AR (+) cells, studies have found that the primary tumor contains AR cells (AR−/low), which exhibit innate resistance to treatment with androgen signaling inhibitors [73,74]. Thus, the presence of these cells impairs the long-term efficacy of drug therapy.

Prostate-specific antigen (PSA) is a prostate-specific differentiation marker expressed only in fully differentiated human prostatic luminal cells. Serum PSA levels can be used for PCa screening and recurrence monitoring. Studies have found that PSA is negatively correlated with tumor progression at the mRNA level, which may be related to the reduced AR signaling during tumor progression [41,75]. Similarly, this association of PSA with tumor progression has been confirmed at the protein level [43]. Increasing evidence confirms that the less differentiated PSA (i.e. no and low expression of PSA [PSA−/low]) AR−/low cell subset in PCa exhibits CSC characteristics (e.g. gene expression profile and epigenetic modification, asymmetric cell division capacity, enhanced DNA damage repair capacity, and tumorigenic ability) similar to those of normal stem cells [41,76]. In patients with CRPC and treatment failure, the PSA−/low AR−/low cell subset is significantly increased and becomes the predominant cell population in the tumor. In addition to the inherent resistance of the PSA−/low AR−/low cell subset to chemotherapy drugs and androgen signaling inhibitors, drug treatment inhibits AR signaling, thereby resulting in the acquisition of a PCSC phenotype by tumor cells [77]. Specifically, long-term ADT enhanced tumor plasticity. Moreover, tumor cells were reprogrammed to express various stem cell markers (e.g. ALDH1A1, CD44, CD133, and SOX2), which are involved in self-renewal, stemness acquisition, and CRPC transformation. It has been demonstrated that the upregulation of the interleukin-6/signal transducer and activator of transcription 3 (IL-6/STAT3) pathway and Wnt pathway after ADT is associated with enrichment of PCSCs. Notably, EMT induced by AR inhibition is also a potential mechanism involved in this process [78]. A recent study showed that ALDHA1 upregulation is involved in PCSC phenotype maintenance, radioresistance, and initiation of bone metastasis, which is associated with increased interaction of ALDHA1 with AR and retinoic acid receptor (RAR) dependent transcriptional programs [79].

#### 3.1.5. TGF-β signaling pathway in PCSCs

TGF-β is a multifunctional cytokine involved in a wide range of physiological processes, such as cell development, tissue homeostasis, tissue regeneration, and immune tolerance [80,81]. It is derived from a wide range of sources in TME. Tumor cells, fibroblasts, macrophages, and platelets can secrete TGF-β. TGF-β interacts with latency associated peptide (LAP) and latent TGF-β binding protein (LTBP) to form an active complex outside the cell. Subsequently, the complex binds to TGF-β receptors on the cell membrane, acting through the Smad-dependent canonical pathway and the Smad-independent non-canonical pathway. Occasionally, the regulatory effects of TGF-β on cancer are contradictory. In early-stage disease, TGF-β signaling induces tumor cell apoptosis and inhibits cancer cell proliferation. At advanced stages of cancer, TGF-β promotes tumor metastasis and progression [82]. Studies have shown that upregulation of the TGF-β pathway leads to tumor metastasis and dissemination by promoting tumor angiogenesis, extracellular matrix deposition, tumor immune escape, and EMT of tumor cells [83,84]. The regulation of EMT by the TGF-β pathway enriches CSCs with high CD44 expression and increases the ability for tumor sphere formation *in vitro* [85]. Consistent with these findings, inhibition of TGF-β signaling reduced the expression of CSC markers This suggested that TGF-β signaling activation contributes to the conversion of non-CSCs to CSCs [86]. Another study revealed that activation of the canonical TGF-Smad3 pathway leads to downregulation of the tumor suppressor speckle type BTB/POZ protein (SPOP) and enhances stemness in PCa [87].

#### 3.1.6. Janus kinase/STAT (JAK/STAT) signaling pathway in PCSCs

The JAK/STAT pathway plays a regulatory role in multiple processes, such as cell proliferation, differentiation, anti-apoptosis, and immune response [88,89]. Binding of ligands (e.g. cytokines) to surface receptors leads to trans-phosphorylation of JAK, which is subsequently phosphorylated to activate STAT. Upon activation, it enters the nucleus as a dimer to regulate downstream molecular signaling [90]. Studies have shown that JAK1/2-STAT3 activation induces cell proliferation and inhibits apoptosis in PCa cells [91]. STAT3 is also involved in CSC maintenance and CSC-mediated tumor metastasis. As mentioned earlier, inhibition of AR signaling caused upregulation of IL-6 expression. Upon binding to its receptors gp80 and gp130, IL-6 activated STAT3, thereby causing expansion of PCSCs [78].

#### 3.1.7. PI3K/AKT signaling pathway in PCSCs

The PI3K/AKT signaling pathway is an important pathway regulating cell cycle, growth, and survival. This pathway plays its regulatory role by activating downstream proteins, such as forkhead box O1 (FOXO1), mTOR, and GSK3. In PCa, upregulation of the PI3K/AKT pathway is associated with androgen-independent signaling, thus inducing ADT resistance and CRPC [92]. In addition, this pathway is associated with the maintenance of PCSCs and the promotion of EMT in PCa cells [93,94]. Studies have shown that inhibition of this pathway can reduce tumor burden, diminish tumor invasiveness, cause the loss of stemness characteristics, and impair the ability of tumor cells to form spheres [94,95].

#### 3.1.8. Other signaling pathway in PCSCs

In addition to the above-mentioned signaling pathways that regulate the survival and stemness of PCSCs, other pathways are also involved in the regulatory process. The NF-κB pathway is highly active in PCSCs. C-X-C motif chemokine ligand 12 (CXCL12) and C-X-C motif chemokine receptor 4 (CXCR4)-mediated activation of NF-κB can significantly enhance the stemness phenotype [96,97]. At present, a variety of NF-κB pathway inhibitors are investigated in preclinical studies or clinical trials. It is thought that the Hippo pathway is also involved in the regulatory process, and its upregulation can promote tumorigenesis and increase the stemness of tumors [98,99]. In addition, evidence has confirmed that glucocorticoid receptors are involved in the functional regulation of PCSCs [100]. Long-term ADT leads to the downregulation of AR, which induces upregulation of glucocorticoid receptors as a compensation. Glucocorticoid receptors can replace AR to regulate the expression of their target genes and negatively regulate the development of PCSCs. MUC1-C has been shown to bind to transcription factor E2F1, activate Brg/Brahma-associated factor (BAF) components and form nuclear complex with them, and promote the self-renewal of PCSCs through Notch1 and Nanog [101]. Galectin-3 (Gal-3) is an extracellular matrix glycan-binding protein, which has been found to affect the tumorigenic and metastatic potential of PCSCs *in vivo* [102,103].

### 3.2. PCSCs and EMT

EMT is the process by which a cell phenotype changes from an epithelial to a mesenchymal phenotype. This process is accompanied by loss of cell adhesion proteins and a change in cell polarity [104]. EMT plays an important role in embryonic development, tissue repair, organ fibrosis, and tumor progression [105]. Increasing evidence shows that the EMT process of tumor cells endows them with migratory ability and CSC characteristics [106,107]. The role of EMT is extensive. In addition to PCa, evidence has confirmed that the EMT process is related to the acquisition of tumor stemness and CSC functional status in a variety of solid tumors, such as breast, pancreatic, and colorectal [24]. The regulatory process of EMT involves a complex interaction network. Moreover, various transcription factors, miRNAs, long non-coding RNAs, post-transcriptional modifications, post-translational modifications, etc., are also involved in the regulation of EMT. Snail family transcriptional repressor 1 (SNAI1), paired related homeobox 1 (PRRX1), FOXC2, and high mobility group AT-hook 2 (HMGA2) act as positive regulators to regulate the EMT process. In contrast, ovo like transcriptional repressor 1/2 (OVOL1/2), grainyhead like transcription factor 2 (GRHL2), zinc finger E‑box‑binding homeobox 1 (ZEB1), etc., act as negative regulators [18,108–110].

With the in-depth study of the EMT process of tumor cells, an increasing number of studies have found that cells in the state of partial EMT often exhibit stronger stemness characteristics than those in a single epithelial phenotype or a single mesenchymal phenotype [111]. A partial EMT state implies that cells have both an epithelial and a mesenchymal phenotype. Therefore, a balance between the cellular regulation of EMT and its opposite, mesenchymal-to-epithelial transition, is required to maintain a mixed state [112]. Loss of FAT atypical cadherin 1 (FAT1) promotes an epithelial phenotype by inhibiting enhancer of zeste 2 polycomb repressive complex 2 subunit-mediated (EZH2-mediated) trimethylation of lysine 27 on histone H3 (H3K27me3) modification and upregulating SOX2. In addition, it promotes a mesenchymal phenotype by regulating the calcium/calmodulin dependent protein kinase II/CD44/SRC (CAMK2/CD44/SRC) axis, resulting in a relatively stable mixed phenotype [113]. It is generally thought that an epithelial phenotype has a higher capacity for tumor proliferation, while a mesenchymal phenotype has a higher capacity for tumor invasion, dissemination, and metastasis. Of note, tumor behaviors and characteristics manifested by cells in different EMT states are not completely consistent in different cancer backgrounds. In mouse models of PCa, it has been observed that mixed or partial EMT phenotypes are linked to higher tumor sphere formation capacity. Notably, mesenchymal cells exhibit higher capacity for tumor initiation, while epithelial phenotypes lead to the greatest metastatic burden [114]. In a skin cancer model, the epithelial phenotype showed higher colony-forming ability, and some EMT states were associated with higher rates of metastasis. However, under different EMT states, tumor cells exhibited similar stemness characteristics. Extensive mechanistic research is necessary to determine whether this difference is caused by tumor heterogeneity or other regulatory factors in the TME.

A partial EMT status of tumor cells is important for establishing distant metastasis [115]. Evidence confirms that tumor cells in a partial EMT state are more likely to metastasize in clusters in the circulation. Compared with single circulating tumor cells, partial EMT cells in clusters have lower ability for tissue penetration, but higher metastatic efficiency. This conclusion was validated in metastatic models of breast and lung cancer [116,117]. The reasons responsible for the high metastatic potential of circulating tumor cells that exist in clusters are unclear. It is generally thought that interactions between polyclonal tumor subsets contribute significantly to this.

### 3.3. PCSCs and TME

TME is a complex ecosystem, which contains a variety of cell types such as tumor cells, immune cells, stromal cells, and other non-cellular components such as cell-secreted factors, growth factors, chemokines, and extracellular matrix [118]. Cellular and non-cellular components in the TME constitute a tightly linked regulatory network, which regulates tumor proliferation, invasion, metastasis and treatment resistance through cell-cell or cell-matrix interactions. TME plays an important role in the stemness maintenance and stemness transformation of CSCs [119].

Tumor associated macrophages (TAMs) refer to macrophages infiltrated in tumor tissues, which can be divided into M1 and M2 macrophages. Generally, M1 macrophages play a role in initiating immune response and anti-tumor, while M2 macrophages play a role in promoting tumor by extracellular matrix remodeling, tumor angiogenesis, and immune suppression. CCL5 has been shown to promote PCSCs self-renewal and PCa metastasis by activating the β-catenin/STAT3 signaling pathway. Knockdown of CCL5 in TAMs significantly inhibited PCa xenograft growth and bone metastasis, as well as the self-renewal and tumorigenicity of PCSCs *in vivo* [120]. Another study has shown the crosstalk between CSCs and TAMs, in which CSC recruit macrophages in TME and convert them into TAMs, which enhance the stemness of PCSCs *via* IL-6/STAT3 pathway [121]. Cancer associated fibroblasts (CAFs) are derived from fibroblasts commonly found in connective tissues, which acquire immunosuppressive and tumor-promoting phenotypes under the action of various stimuli in cancerous conditions and are called CAFs. In solid tumors, CAFs have been found to be widely involved in tumor progression, tumor angiogenesis, CSC niche construction, and extracellular matrix remodeling. Studies have found that IL-6 derived from PCa cells promotes the activation of CAFs, and the activated CAFs secrete metalloproteinases to promote the EMT of tumor cells, thereby enhancing tumor stemness [122]. A recent study has shown that upregulation of fibroblast monoamine oxidase A in the tumor stroma induces the tumorigenic phenotype of fibroblasts and promotes PCa cell proliferation and stemness marker expression of PCSCs through the IL-6/STAT3 pathway [123]. Subsequent studies have found that monoamine oxidase B plays a similar role in stromal cells, but its effect on the stemness maintenance of PCSCs has not been clarified [124]. Myeloid-derived suppressor cells (MDSCs) are another type of immunosuppressive cells that promote tumor growth and have been implicated in the stemness maintenance of PCSCs [125]. In addition to these cells, a variety of cell types, such as dendritic cells, natural killer cells, T cells, endothelial cells and adipocytes, have been proved to be closely related to the stemness regulation of CSCs in solid tumors or hematological malignancies [126,127].

Bone is one of the most common sites for metastatic PCa. Contrary to previous understanding, emerging evidence suggests that cancer metastasis can occur in the early stages of the disease rather than exclusively in the late stages [31]. The distant metastasis of tumor cells is a complex and highly regulated process [128]. As previously mentioned, the EMT of the primary tumor facilitates the metastasis of tumor cells. This population of metastasis-initiating cells enters the bloodstream and subsequently colonizes suitable organs. In this process, tumor cells present in the bloodstream are referred to as circulating tumor cells (CTCs), some of which exhibit characteristics of CSCs, potentially due to varying degrees of EMT [95,129]. Research has confirmed that CSC markers are upregulated in CTCs across a range of malignancies, including PCa [130]. This observation may also elucidate the chemoresistance of CTCs reported in clinical studies [131]. The selection of tumor colonization sites is not random. Prior to metastasis, the proposed metastatic sites are regulated to establish a premetastatic microenvironment that facilitates the extravasation and retention of metastatic cancer cells [132]. The quiescence and activation of metastatic cells at distant metastatic sites are regulated and supported by the metastatic microenvironment. For example, growth arrest-specific protein 6 (GAS6), tyrosine protein kinase MER, TGF-β2, bone morphogenetic protein 7 (BMP7) and other factors regulate the quiescence and activation process of PCa bone metastasis [133–135]. Meanwhile, tumor cells also contribute to the remodeling of bone metastasis microenvironment by regulating the balance of osteogenesis and osteoclast, tumor angiogenesis, extracellular matrix secretion, and immunosuppression [136,137].

Currently used cell lines and animal xenograft models are difficult to simulate the real human TME. The emergence of new research models, such as humanized mouse models and organoids, will facilitate the in-depth exploration of the interaction mechanism between human tumors and TME [138,139].

## 4. Targeted therapy strategies for PCSCs

**Figure 2.**
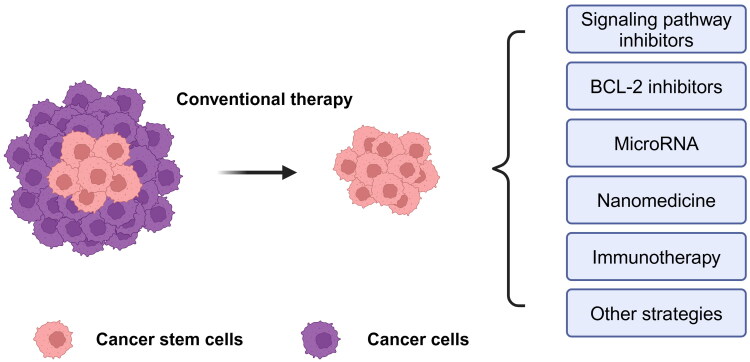
Targeted therapy strategies of PCSCs. PCSCs are tolerant to conventional treatments such as androgen signaling inhibitors, chemotherapy, and radiotherapy. A variety of therapeutic strategies targeting PCSCs have emerged, such as signaling pathway inhibitors, BCL-2 inhibitors, MicroRNAs, nanomedicines, and immunotherapy (Created with bioRender.com).

The innate resistance of PCSCs to treatment with androgen signaling inhibitors and chemoradiotherapy allows this small population of cells to survive the therapeutic stress. CSCs that survive the treatment have the ability to restart the tumor, leading to the recurrence, progression, and dissemination of PCa. According to previous studies, the abundance and functional status of PCSCs are regulated by multiple factors, including the cells themselves and the surrounding TME, which form an intricate interactive regulatory network [140]. This process also provides a potential therapeutic target for PCSCs (as shown in Figure 2). In this section of the review, we introduce in detail the currently available drugs targeting PCSCs with potential clinical value from the aspects of signaling pathway inhibitors, miRNAs, nanomedicine, and immunity.

### 4.1. Signaling pathway inhibition and PCSC-targeted therapy

#### 4.1.1. Wnt/β-catenin signaling pathway inhibition

Wnt secretion inhibitors, such as IWP, Wnt-C59, LGK974 (Wnt974), and ETC-159, inhibit Wnt secretion by acting on the key enzymes (porcupine) of Wnt protein palmitoylation [141,142]. Anti-tumor effects of LGK974 and ETC-159 have been observed in preclinical studies of colorectal cancer, pancreatic cancer, renal cancer, PCa, etc. [143–145]. Other drugs that target the Wnt pathway include the Wnt pathway functional antagonists, as well as drugs that target Wnt receptor interactions, inhibit dishevelled activation, stabilize the destruction complex, and target β-catenin partners [53]. Clinical studies have shown that the Wnt pathway antagonist Dickkopf 3 (DKK3) induces inhibition of tumor growth in patients with localized or metastatic PCa [146]. Foxy-5 is a formylated amino acid fragment that acts as a Wnt-5a mimic to activate the receptor and inhibit the invasion and migration of epithelial cancer cells. It has also exhibited a good safety profile in a phase I clinical trial (NCT02020291). In addition, rottlerin, salinomycin, niclosamide targeting the LDL receptor related protein 6 (LRP6), and triptolide targeting β-catenin can also promote the apoptosis of PCa cells [147–150]. In general, although various Wnt pathway inhibitors have shown some clinical value, there is a lack of strong evidence to further confirm the efficacy and safety of these drugs in patients with PCa. Thus, further clinical studies are required [151,152].

#### 4.1.2. Notch signaling pathway inhibition

Common Notch signaling inhibitors can be divided into three categories: monoclonal antibodies targeting Notch receptors (brontictuzumab, tarextumab, etc.); γ-secretase inhibitors (GSIs; PF-03084014, RO-4929097, JSMD194, etc.); and small molecule inhibitors of the Notch transcription complex (CB-103, SAHM1, IMR-1, etc.) [153]. Among them, GSIs inhibit Notch nuclear translocation by inhibiting Notch intracellular domain cleavage. GSIs have shown anti-tumor activity in a variety of advanced solid tumors, such as lung and breast cancer [154]. In patients with chemotherapy-resistant CRPC, GSIs play an important role by inhibiting the proliferation of CSCs and restoring tumor sensitivity to chemotherapy drugs/ADT [155,156]. Studies have shown that the combination of GSIs with chemotherapy drugs produces better therapeutic responses than GSIs alone. This synergistic effect has also been observed following the combination of GSIs with ADT [157–160]. This evidence indicates the potential value of GSIs as an adjunct to ADT. This approach may provide an even greater therapeutic benefit for patients with PCa in the future. Although GSIs has shown promising therapeutic potential in preclinical models, the extensive Notch signaling blockade produced by GSIs has raised concerns regarding drug safety. Of note, serious adverse effects caused by GSIs include gastrointestinal toxicity, lymphoid tissue abnormalities, and development of skin cancer [161,162]. At present, researchers are exploring new drugs selectively targeting γ-secretase [163]. Furthermore, specific antibody-based targeting strategies also attract considerable research attention. It has been found that a specific antibody targeting Notch1 (OMP-A2G1) can inhibit tumor growth by affecting DNA damage repair and AR expression in a mouse model of PCa [164].

#### 4.1.3. Hh signaling pathway inhibition

Hh signaling inhibitors are mainly divided into SMO receptor inhibitors and GLI transcription factor inhibitors [165]. Hh inhibitors commonly used in the treatment of PCa include sonidegib (LDE-225), vismodegib (GDC-0449), erismodegib, cyclopanmine, and GANT-61. The first two of these agents have been approved by the US Food and Drug Administration for the treatment of metastatic or locally advanced basal-cell carcinoma. In preclinical studies, it has been demonstrated that Hh inhibitors exert a wide range of anti-tumor effects [166]. However, in two previous clinical studies, sonidegib and vismodegib did not induce a significant therapeutic response in patients with high-risk localized PCa and metastatic CRPC [167,168]. This evidence suggests that Hh inhibitors alone are not an appropriate treatment option for patients with advanced PCa. SMO-independent pathway activation may be partly responsible for the failure of SMO receptor inhibitors to achieve the desired therapeutic effect. This is supported by the comparative efficacy study of GANT-61 and GDC-0449. The results showed that GANT-61 was more effective than GDC-0449 in inhibiting PCa cell proliferation, tumor sphere formation, and CSC marker expression. These data suggested that the inhibition of GLI, a downstream molecule of the Hh pathway, exerts a greater anti-tumor effect [169]. In addition, the combination of Hh signaling inhibitors with chemotherapy or other drugs is also a promising treatment strategy. Nevertheless, it is necessary to examine whether this combination therapy will lead to additional drug toxicity [170–172]. Itraconazole is a common anti-fungal agent that also has SMO inhibition. Itraconazole has shown PSA response and PSA progression-free survival benefits in patients with biochemically recurrent and metastatic CRPC in clinical trials [173,174]. In addition, miRNA-361-3p, genistein and darinaparsin also showed inhibition of Hh pathway in preclinical studies [175–177].

#### 4.1.4. Inhibition of other signaling pathways

PI3K/AKT pathway inhibitors, such as pilaralisib (XL-147), BEZ235, and buparlisib; NF-κB pathway inhibitors, such as bortezomib and eupatilin; as well as TGF-β inhibitors and antiangiogenic agents (cetuximab) have been associated with varying degrees of tumor inhibition and stemness loss in basic and clinical studies [178–180]. In addition, salinomycin and protein kinase Cα (PKCα) can inhibit the stemness transition process of CSCs by targeting tumor cells with a mesenchymal phenotype [181]. Studies have demonstrated that telomerase inhibitors (imetelstat), ABC transporter inhibitors (dofequidar fumarate), and metformin target CSCs [182]. Inhibition of retinoic acid signaling can lead to CSC necrosis, so RAR inhibitors (e.g. AGN194310, AGN194431, miRNA-30a-5p) and blocking retinoic acid synthesis can be potential CSC treatment options [183–185].

#### 4.1.5. B-cell lymphoma-2 (BCL-2) inhibitors

BCL-2 is an anti-apoptotic protein, and preclinical studies have shown increased expression of BCL-2 in a variety of malignancies, including PCa [186]. Furthermore, BCL-2 expression is higher in CRPC than in treatment-naïve PCa, implying that BCL-2 may be involved in the development of resistance during ADT or chemoradiotherapy [187,188]. In previous studies, BCL-2 has been identified as a key target for targeting PCSCs [74]. Thus far, numerous BCL-2 inhibitors have been discovered, such as ABT-101, ABT-199, ABT-263, ABT-737, etc. Clinical trials have reported the therapeutic effect of BCL-2 inhibitors in patients with PCa [189,190]. Early studies of ABT-101 combined with docetaxel and prednisone (ADP) for the treatment of patients with metastatic-CRPC have shown an increased incidence of adverse events and no additional survival benefit compared with docetaxel and prednisone (DP) alone (median overall survival: 18.1 vs. 17.8 months, respectively; hazard ratio = 1.07, 95% confidence = 0.72–1.55 [191]. A subsequent study investigated the response of patients with newly diagnosed metastatic hormone-sensitive PCa to treatment with AT-101 plus ADT. Unfortunately, the PSA response rate observed with the combination regimen was significantly lower than anticipated (31% vs. 68%) [192]. A growing body of evidence demonstrates that BCL-2 inhibitors exert synergistic effects with tyrosine kinase inhibitors and immunotoxins, thereby providing theoretical support for the clinical application of combination therapy [193–196].

### 4.2. miRNAs

The term miRNAs refer to a class of non-coding RNA molecules that play a regulatory role in tumorigenesis, cell proliferation, differentiation, and apoptosis[197]. According to previous studies, the inhibitory effect of miRNA-200 on LIN28 relieved the repression of LIN28 on the Let-7 miRNA precursor [198]. Members of the Let-7 miRNA family can directly target the 3’untranslated regions of several self-renewal genes, such as HRAS, HMGA2, and OCT4. In PCa, miRNA-200b/c decreased tumor initiation and impaired stemness maintenance. In breast cancer, miRNA-200c inhibited the EMT process mediated by SNAI1, TWIST1, and TGF-β, thereby inhibiting the generation of CD44 + CD24− CSCs. In PCa, the roles of miRNA-34a and miRNA-141 in the regulation of EMT have also been extensively studied [199,200]. In addition, miRNA-143 and miRNA-145 inhibited the activity of PCa bone metastasis cells and impaired their ability for colony formation by inhibiting the expression of CSC markers and stemness factors [201]. The miRNA-148a and miRNA-152-3p can synergistically inhibit the expression of stemness markers, such as SOX2 and OCT4, inhibit tumor migration and invasion, and promote cell apoptosis. Recent studies have provided a new miRNA therapeutic strategy for PCa. The miRNA-7 regulates the process of glycolysis in tumor cells by regulating key pathways, such as hypoxia-inducible factor-1α (HIF-1α), thereby reshaping the acidic TME and inhibiting tumor cell growth [202]. Another result showed that CAFs exosomes drive PCa metastasis through the miRNA-500a-3p/FBXW7/HSF1 axis in hypoxic microenvironment, which may be related to the stemness regulation of miR-500a-3p [203]. Interestingly, evidence suggests that the upregulation and downregulation of several miRNAs may promote lineage transformation in PCa, leading to the development of neuroendocrine PCa [204]. In-depth exploration of miRNAs regulating the function and state of PCSCs can provide new therapeutic targets or diagnostic tools, which may be helpful for the treatment and risk stratification of patients with PCa [205].

### 4.3. Nanomedicine

With the rapid development of nanotechnology in recent years, the potential utilization of nanomedicine in tumor treatment has attracted extensive attention from researchers and clinicians [206]. Compared with conventional drugs, nanomedicine offers better pharmacokinetics and higher efficiency for drug delivery [207,208]. Off-target effects are also reduced, indicating that nanodrugs are associated with an improved safety profile and a wider drug dose window. Another important feature of nanomedicine is its tumor-targeting ability, which can be divided into passive and active targeting. Passive targeting is due to the ‘enhanced permeability and retention effect’ caused by tumor vascular congestion and peripheral lymphatic drainage disorders. This passively enriches the nanomedicine in the tumor area and exerts a cytotoxic effect on both tumor cells and normal cells [209]. Therefore, passive targeting is not a strictly tumor cell-selective option. Active targeting refers to the selective delivery of drugs loaded in nanocarriers. Ligands or specific antibodies on the surface of the nanocarrier permit binding to the corresponding receptor or antigen of specific tumor cells. This increases the efficiency of drug uptake by tumor cells and reduces the impact on surrounding normal tissues.

A variety of natural plant extracts possess anti-tumor activity, and studies have confirmed that this therapeutic effect is achieved through effects on CSCs. In PCa, the use of nanoparticles loaded with curcumin and silibinin increased the metabolic stability and bioavailability of the drug, as well as improved the anti-cancer effect [210,211]. In addition, the selective delivery of various types of drugs, such as nucleic acids (DNA, miRNAs, siRNA, etc.), proteins, peptides, and lipid-soluble drugs can be realized using liposomes, polymeric nanomicelles, polymeric nanoparticles, and nanogels as drug carriers. At present, multidrug combination is a popular treatment strategy, and the use of nanocarriers can facilitate the synergistic effect of drugs. The combination of traditional chemotherapy drugs with CSC inhibitors appears to be an ideal treatment plan. Previous studies have reported simultaneous loading of nanocarriers with Hippo pathway inhibitors (verteporfin), vascular-disrupting agents (combretastatin), and chemotherapeutic agents. This strategy has been associated with satisfactory therapeutic responses in models of triple-negative breast cancer [212]. In another study, researchers used a nanocarrier loaded with a magnetic core and a chemotherapeutic drug in addition to a specific antibody for targeting lung CSCs. This targeted therapy induced a significant cytotoxic effect on CSCs *in vitro* and *in vivo* with good safety [213]. Interestingly, the sequential release of multiple drugs can be achieved by clever design of delivery vectors. In a mouse model of breast cancer, hypoxia within the tumor induces the release of all-trans retinoic acid, which promotes the differentiation of CSCs and increases the levels of intracellular reactive oxygen species. This increase in reactive oxygen species induces the release of camptothecin from nanoparticles, which kills differentiated CSCs and prevents tumor recurrence and metastasis [214].

At present, numerous nanomedicines targeting CSCs are under investigation. Nanomedicine has been linked to promising therapeutic prospects in a variety of solid tumors [215]. Nevertheless, this approach is currently not employed in the treatment of PCa. Hence, further research is required. In addition, the selection of cell-specific targets, stability in circulation, and ability for drug release in tumor cells remain major challenges for the application of nanomedicine [209,216].

### 4.4. Other therapeutic strategies targeting PCSCs

Our previous review discussed the research progress regarding immunotherapy for patients with advanced PCa [217]. Most of these drugs or immune products target non-stem tumor cells. With the deepening understanding of CSCs, immunotherapy targeting CSCs appears to be a promising option [218]. Studies revealed that CSCs have the ability of immune evasion, thereby evading the cytotoxic effect of natural killer cells and CD8+ cytotoxic T cells. This is achieved by directly inhibiting immune cells or specific immune response factors, inducing programmed cell death of T lymphocytes, etc. [219]. A variety of immunotherapy strategies targeting CSCs have been developed, including cytokine-induced killer cells, natural killer cells, CD8 + T lymphocytes, dendritic cells, oncolytic virotherapy, and CAR-T therapy [220,221]. In preclinical studies, chimeric antigen receptor-T (CAR-T) therapy targeting epithelial cell adhesion molecule (EpCAM) has shown significant cytotoxic ability against PCSCs [222]. Similarly, another study used CD44 and EpCAM-sensitized dendritic cells to activate cytokine-induced killer cells, which showed potent cytotoxicity against PCSCs *in vitro* and *in vivo* [223]. These data highlighted the potential clinical value of immune strategies for targeting PCSCs. Undeniably, there are several challenges that need to be overcome in immunotherapy [224]. Firstly, the identification and selection of CSC markers is a major task in this setting. CSCs often lack specific surface antigens due to the low degree of differentiation; this complicates the selection of suitable targets. Secondly, PCa is considered a ‘cold’ tumor lacking immune infiltrating cells. Moreover, the surrounding environment is rich in immunosuppressive cells and suppressor factors. These facts may lessen the anti-tumor effect of immune agents [225,226]. In addition, significant tumor heterogeneity indicates that the treatment strategy of immunotherapy may need to be tailored to specific patients. This limits the utilization of this therapeutic approach in patients with cancer. Customized immune agents are also associated with an unbearable treatment burden.

Considering the complexity of CSC regulatory networks, other potential targeting strategies are also being explored. For example, long non-coding RNAs and circular RNAs have been associated with the self-renewal, stemness transformation, and chemoresistance of CSCs [227,228]. Induced differentiation can also be used to target CSCs. In addition to all-trans retinoic acid, vitamin D and histone deacetylase inhibitors can be used to promote differentiation [229]. The TME provides a suitable environment for tumor growth and development. Similarly, the survival and stemness maintenance of CSCs also require the support of environmental factors, termed CSC niche. TAMs, CAFs, mesenchymal cells, extracellular matrix, and hypoxic environment in the niche are closely related to the functional status of CSCs, thus providing a series of potential therapeutic targets [120,230]. The metabolic regulation of CSCs is another focus of research on cancer therapy [231]. For example, the balance between oxidative phosphorylation and glycolysis, and the enrichment or deficiency of metabolites may affect CSCs [232,233]. Lastly, the combination of monoclonal antibodies targeting biomarkers of PCSCs with radiotheranostic agents is also a therapeutic strategy currently being explored [234].

## 5. Limitations and future perspectives

The functions and therapeutic applications of CSCs have become significant research focal points in recent years. An increasing number of researchers are exploring the considerable potential contained within this small group of cells. However, challenges in the isolation and identification of CSCs persist, and the definition of the CSC population is continually evolving. Studies have shown that some CSCs do not express established markers, while certain non-CSCs do express these markers, thereby complicating the processes of isolation and identification. Limiting dilution assay, colony/sphere formation assay, fluorescence-activated cell sorting (FACS) and magnetic-activated cell sorting (MACS) are widely used to isolate and identify CSCs. The selection of specific surface markers according to the specific tumor type and genotype will improve the efficiency of cell discrimination [31].

Some evidence for CSCs has been derived from cell lines and tumor xenograft animal models, which may have innate limitations, for example in genetic heterogeneity and tumor niches [235]. Organoids are a promising three-dimensional *in vitro* model [236]. Compared with cell lines and animal models, organoids can maintain the characteristics of parental cancer cells and simulate tumor-stroma interaction to reproduce the human TME. Whole exome sequencing and RNA sequencing confirmed the similarity of the PCa organoids to the parental cancer [237]. Organoids derived from patient tumor tissues preserve individual-specific tumor characteristics and have been widely utilized in studies of tumor progression, invasion, and the prediction of therapeutic efficacy [139,238]. Tumor xenograft models are one of the most common models in the study of CSCs. Orthotopic inoculation of tumor cells can better reflect the invasiveness of tumors. Xenograft models are constructed based on immunodeficient animals, and it is insufficient to evaluate the therapeutic effect of immune agents on CSCs [239]. To compensate for this deficiency, humanized mouse models have emerged, in which mice are endowed with the human immune system by transplanting human immune organs or immune cells [240,241]. Although the current humanized mouse model cannot fully replicate the human immune system, it offers an innovative platform for researchers to investigate the mechanisms of tumor immunity [242]. It is important to note that although organoid models and patient-derived xenograft models have increasingly contributed to CSC research, both models still exhibit some limitations that require improvement. For instance, the absence of vascular structures in in organoid models may hinder their maturation. Additionally, long-term passage of organoids can lead to a loss of genetic heterogeneity, and the lack of immune cells in organoid models further complicates their utility [243,244]. Patient-derived xenograft models face challenges such as extended modeling times, low success rates, and the potential loss of genetic characteristics from the parental tumors [245]. Furthermore, both organoid and patient-derived xenograft models do not exclusively support the growth of CSCs, making it difficult to differentiate between CSCs and proliferating cancer cells. Continuous improvement and innovation in preclinical models are essential for understanding the complex interactions between CSCs and other cellular components in TME. In recent years, the potential applications of immune cell and tumor organoid co-culture technology, microfluidic organoid chip technology, and other emerging technologies have garnered significant attention [246,247].

For PCSCs, there is an urgent need for a more detailed description of their characteristics and search for specific molecular markers. Furthermore, a thorough understanding of the crosstalk between PCSCs and cellular and non-cellular components of the TME is of great significance for tumor dissemination and metastasis. Specifically, the process by which a small number of circulating tumor cells establish distant metastasis is particularly important [248].

Overall, targeting CSCs provides a promising therapeutic strategy for patients with PCa, especially those with CRPC and chemotherapy-resistant PCa. This approach has shown promising results in a number of preclinical studies. Additional clinical studies are warranted to further verify the safety and efficacy of targeted PCSC therapy [249]. In the future, the continuous development of new technologies, such as next-generation sequencing technology and various omics, may provide useful tools for the screening and identification of CSCs [250,251]. The exploration of new CSC markers and regulatory pathways may provide more target options for cancer therapy [252]. Importantly, the use of artificial intelligence and nanoinformatics might facilitate the design of nanomedicine particles characterized by good circulatory stability and efficient tumor-specific delivery [253–255]. Combination therapy is one of the most promising treatment options for cancer [256]. The combination of CSC-targeted drugs with other traditional agents may simultaneously kill CSCs and common tumor cells. Further research on this combination regimen is necessary in the future to determine its safety profile and identify the most appropriate patient population for this treatment.

## 6. Conclusion

The CSC theory provides a reasonable explanation for tumor heterogeneity and plasticity. PCSCs can survive or be enriched in patients receiving ADT and chemoradiotherapy, as well as mediate subsequent tumor recurrence and progression. A deeper understanding of PCSCs may assist in identifying potential CSC targets. Although the complex regulatory network of PCSCs remains partly understood, exploratory studies have shown surprising clinical application potential for targeting PCSCs. We expect that this strategy will provide therapeutic options for patients with PCa in the future.

## Supplementary Material

Figure.docx

## Data Availability

Data sharing is not applicable to this article as no new data were created or analyzed in this study.
