# Supplementary material for: Stemness regulation in prostate cancer: prostate cancer stem cells and targeted therapy
Source: Ann Med. 2024 Dec 23;57(1):2442067. doi: 10.1080/07853890.2024.2442067 (PMC11703425; doi:10.1080/07853890.2024.2442067)
Supplement: Figure.docx [file IANN_A_2442067_SM4079.docx]

**Figures:**


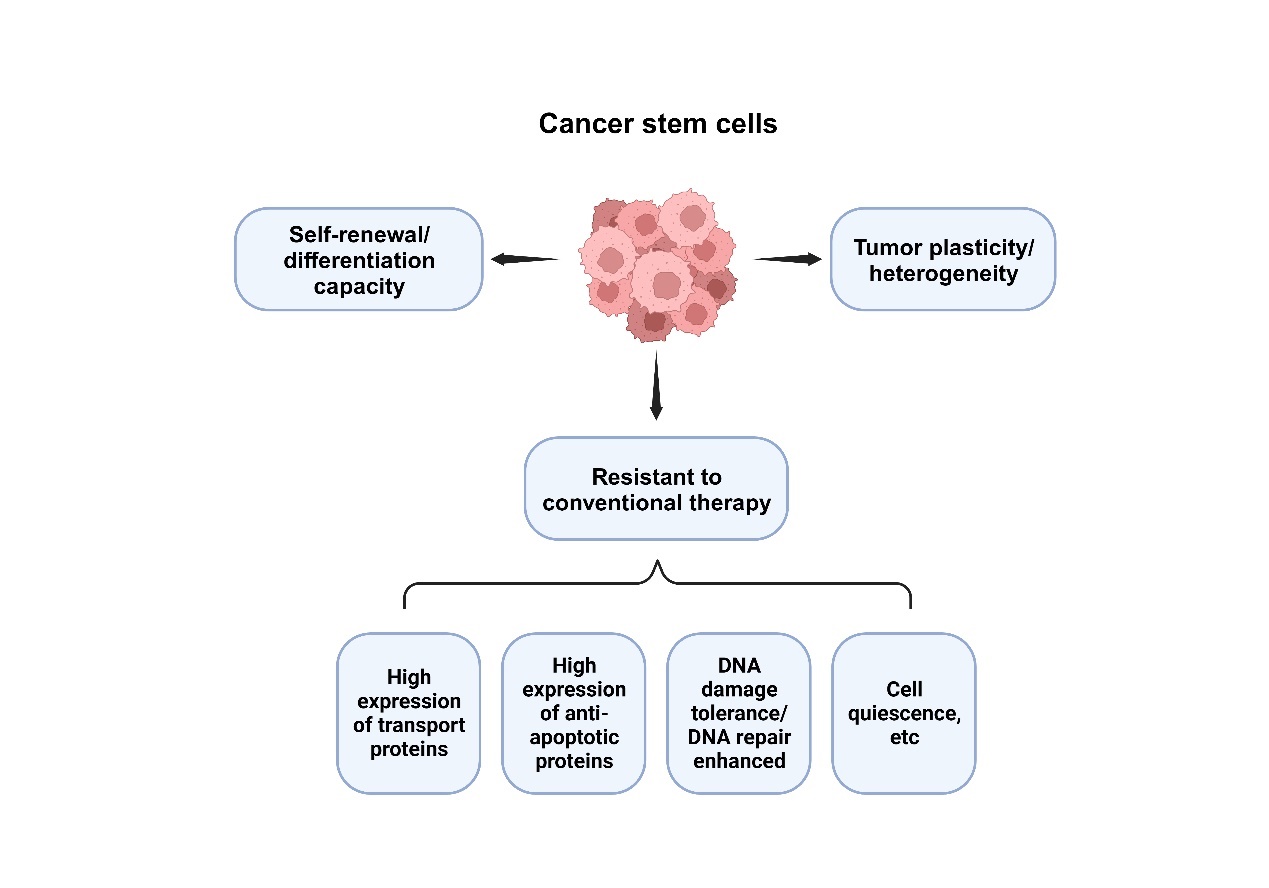


Figure-1: Characteristics of CSCs

CSCs are a subpopulation of cells that are ubiquitous in tumors and have similar characteristics to normal stem cells, namely, self-renewal and differentiation ability. CSCs are a group of dynamically changing cell subsets that can achieve the transformation of stemness and non-stemness under the action of regulatory factors, which reflects the plasticity and heterogeneity of tumors. In addition, compared with common tumor cells, CSCs are more resistant to conventional treatment strategies, which is related to the expression of high levels of transport proteins and anti-apoptotic proteins, enhanced DNA damage tolerance and DNA repair ability, and relative quietness of cells. (Created with bioRender.com)


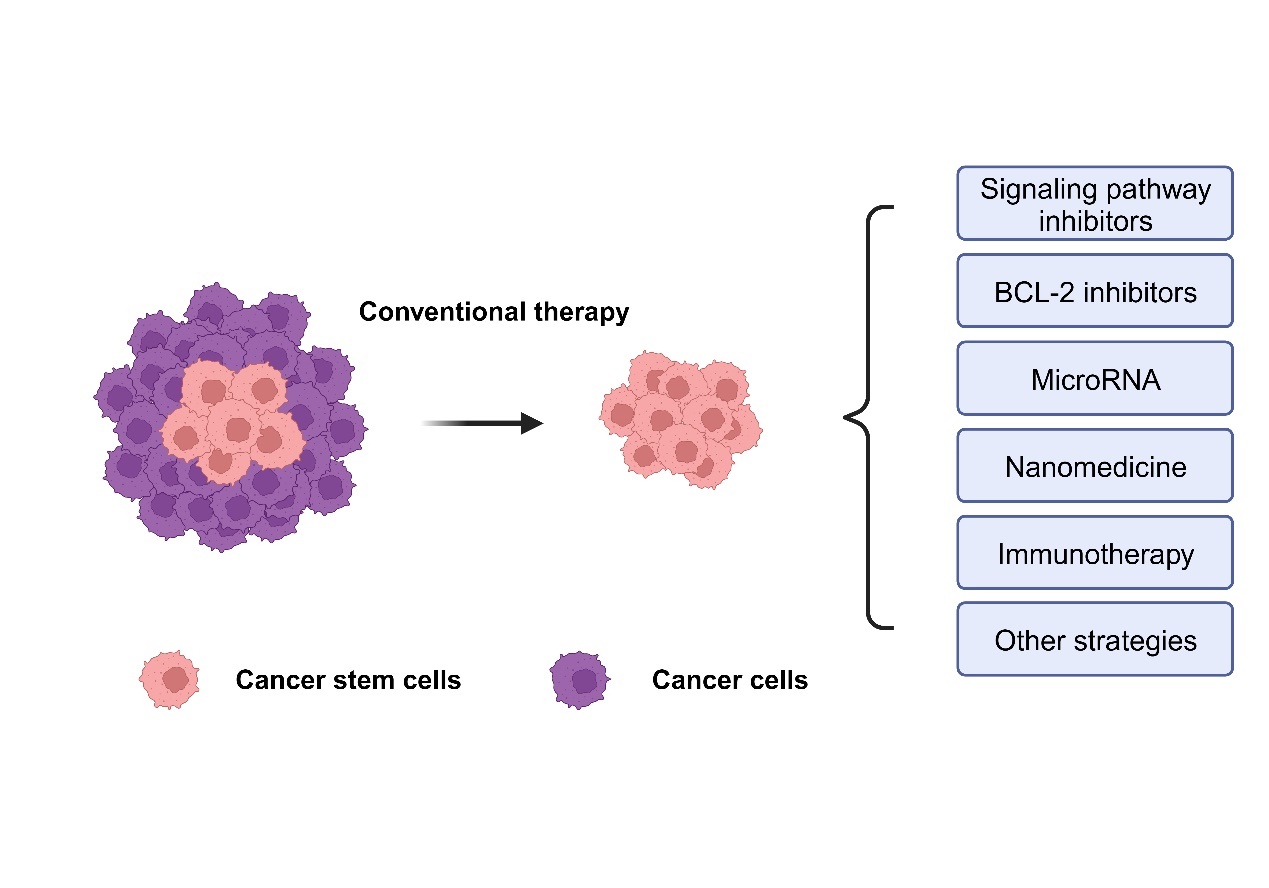


Figure-2: Targeted therapy strategies of PCSCs

PCSCs are tolerant to conventional treatments such as androgen signaling inhibitors, chemotherapy, and radiotherapy. A variety of therapeutic strategies targeting PCSCs have emerged, such as signaling pathway inhibitors, BCL-2 inhibitors, MicroRNAs, nanomedicines, and immunotherapy. (Created with bioRender.com)
